# Analysis of Sport Supplement Consumption and Body Composition in Spanish Elite Rowers

**DOI:** 10.3390/nu12123871

**Published:** 2020-12-18

**Authors:** Raúl Domínguez, Rubén López-Domínguez, Álvaro López-Samanes, Pol Gené, José Antonio González-Jurado, Antonio Jesús Sánchez-Oliver

**Affiliations:** 1 Escuela Universitaria de Osuna (Centro Adscrito a la Universidad de Sevilla), 41640 Osuna, Spain; rauldh@euosuna.org; 2 Departamento de Educación Física y Deporte, Universidad de Sevilla, 41013 Sevilla, Spain; 3 Studies Research Group in Neuromuscular Responses (GEPREN), University of Lavras, Lavras 37200-000, Brazil; 4 Facultad del Deporte, Universidad Pablo Olavide, 41013 Sevilla, Spain; rubenlopdom@gmail.com (R.L.-D.); polgene@gmail.com (P.G.); jagonjur@upo.es (J.A.G.-J.); 5 Exercise Physiology Group, Faculty of Health Sciences, School of Physiotherapy, Universidad Francisco de Vitoria, 28223 Madrid, Spain; alvaro.lopez@ufv.es; 6 Federación Española de Remo, 28008 Madrid, Spain; 7 Departamento de Motricidad Humana y Rendimiento Deportivo, Universidad de Sevilla, 41013 Sevilla, Spain

**Keywords:** elite athlete, anthropometry, ergogenic aids, sport performance

## Abstract

The aim of this study was to analyze the anthropometric characteristics and sport supplement (SS) consumption patterns of heavyweight and lightweight international rowers. Methods: The 13 heavyweights (11 males) and seven lightweights (five males) of the Spanish National Rowing Team were recruited for the study. Body composition was measured by bio-impedance analysis, and the questionnaire used in this investigation was previously validated to assess SS consumption. According to anthropometrics parameters, it was reported that male heavyweight rowers were heavier (*p* < 0.001) and taller (*p* < 0.001), but no statistical differences were reported for % body fat (*p* = 0.104) or % lean body mass (*p* = 0.161). All rowers reported consumption of at least one SS. Based on the Australian Institute of Sport’s classification, higher medical supplement consumption was observed when comparing heavyweight rowers to lightweight rowers (2.5 ± 1.1 vs. 1.7 ± 0.5, *p* = 0.040). There were no differences in the totals of group A (strong scientific evidence for sports scenarios, *p* = 0.069), group B (emerging scientific support, deserving of further research, *p* = 0.776), or group C (scientific evidence not supportive of benefit and/or security amongst athletes, *p* = 0.484). The six most consumed SSs were iron (85%), caffeine (85%), β-alanine (85%), energy bars (85%), vitamin supplements (80%), and isotonic drinks (80%), with no statistical differences between heavyweight and lightweight rowers (*p* > 0.05). These results suggest that the absence of differences in body composition (expressed as a percentage) do not represent anthropometric disadvantages for heavyweight rowers. In addition, SS consumption was similar between rowers, reporting only higher medical supplement consumption in heavyweight rowers.

## 1. Introduction

Elite athlete status is a partially heritable trait, along with other physiological, anthropometrical, and psychological traits that contribute to elite sports performance [1]. These physical and physiological capabilities differ depending on the sport practiced [2]. Consequently, the activity’s specificity must be taken into account in the training of elite athletes [3]. As previously reported, a 0.6% performance improvement today is sufficient to make a difference in sports performance [4].

Rowing is one of the oldest Olympic sports, having been represented since the first modern Olympic games in Athens in 1896. Rowing athletes compete in different modalities that cover 2000 m in sculling or sweep styles, and are divided into heavyweight and lightweight divisions. Heavyweight rowers can be any weight. Lightweight men must be below 72.5 kg or part of a 70 kg crew average, and lightweight women must weigh below 59 kg or have an average crew weight of 57 kg [5]. Rowing competitions last between 5.5 and 8 min, and have an approximate mean velocity between 5.3 and 6.0 m/s. In the metabolic domain, the oxidative metabolism contribution is around 67%, while glycolysis and ATP-PCr contributions, respectively, represent about 21% and 12% [6]. Thus, rowers’ performance requires a mixture of highly developed aerobic and anaerobic systems [5,7]. Performance has been shown to correlate with VO_2max_ [8,9], metabolic power developed at intensities around 4 mmol·L^−L^ blood lactate concentration [8], and mean and peak power obtained in a maximal test lasting for 30 [9] or 40 s [10].

At the anthropometric level, rowing athletes are characterized by high values of height, weight, strength, and power output. They also have low values of body fat that differ depending on the competitive modality [1,11]. Heavyweight rowers are generally taller and heavier [12], with higher levels of body fat [5], which enables them to obtain higher power outputs during a 2000 m test when compared to lightweight rowers (463 vs. 405 W) [13]. However, when these values are related to body mass, lightweight rowers present higher values of mean power in relation to body mass when compared to heavyweight rowers (5.1 W/kg vs. 4.2 W/kg) [14]. In addition, female rowers reported higher power values comparing heavyweight rowers versus lightweight rowers (310 W vs. 290 W), therefore, higher values are reported in lightweight rowers relativized to body mass compared to heavyweight (4.6 W/kg vs. 3.9 W/kg) [12]. Due to the importance of weight in this sport’s modalities, a continuous control of body weight is required [5,15], especially in lightweight rowers who not only undergo chronic restriction of energy intake throughout the year, but also aggressive weight loss strategies to compete within the weight limits established by the World Rowing Federation (FISA). These weight restriction practices may have a negative effect on rowing performance [5,6,7,15].

Thus, lightweight rowers in particular experience frequent episodes of body weight loss, leading to a high incidence of eating disorders [12,15]. This increases the risk of developing energy and nutrient deficits [5], increasing the risk of injuries and health impairment, reducing training intensities during training/competitions, and affecting exercise adaptations and sports performance goals [5,6,7,15]. According to the information previously mentioned, Slater et al. (2014) stablished a nutrition education program to assist rowers in achieving specified goals [12].

The inclusion of sport supplements (SSs) is recommended within the competitive nutrition plans of elite rowers to increase training adaptations [5] and prevent nutritional deficits [16]. Previous investigations have reported that the consumption of SSs is widely practiced in the majority of international athletes, showing that the use of SSs in international athletes is higher than that of national athletes [17,18,19]. However, to our knowledge, no studies have assessed the prevalence of SS consumption in heavyweight and lightweight rowers. Thus, the primary aim of this study was to analyze the anthropometric characteristics and SS consumption of different rower categories (heavyweight vs. lightweight) in international rowing athletes. The second aim was to analyze the consumption of SSs in the different categories and groups based on the level of evidence recently established by the Australian Institute of Sport [20].

## 2. Materials and Methods

### 2.1. Participants

All members of the Spanish National Rowing Team (age: 23 ± 3 years) participated, including 13 (11 males) heavyweight rowers and seven (five males) lightweight rowers. The participants, who were all at least 18 years old, gave their informed consent after receiving verbal and written explanations of the study. The study obtained the approval of the Spanish Rowing Federation (FER), and was conducted in accordance with the Declaration of Helsinki. The study was approved by a formally constituted ethics committee (Alfonso X El Sabio University).

### 2.2. Procedure

The study was carried out at Seville’s Specialized High-Performance Centre (C.E.A.R.) of Rowing and Canoeing during the competitive season. Weight was measured with a calibrated balance beam scale (Seca 700, Hamburg, Germany) under a standardized method (fasting, without shoes, and minimal clothing). Height was measured with a telescopic height rod (Seca 220, Hamburg, Germany). The body composition of the subjects was obtained using a tetrapolar electrical bioimpedance meter (BIE) (50 Hz; Bodystat 1500, Bodystat Ltd., Isle of Man, UK), with its software having demonstrated a good accordance with the anthropometry [21] and dual-energy absorptiometry assessment [22]. Data collection followed the protocol and standards established by Lukaski et al. [23] and the Declaration of the Spanish Group of Kinanthropometry [24], taking into account the considerations of Sergi et al. [25]. The body composition variables scrutinized were weight (kg), height (m), body mass index (BMI) (kg/m^2^), body fat (kg), lean body mass (kg), fat mass (%), and lean body mass (%).

Participants completed a questionnaire about SS consumption that was previously validated [26]. This questionnaire, used previously in studies involving elite athletes from different sports modalities [27,28,29,30,31], is organized into three parts: personal and social data of the participant (i); sports activity and its contextualization (ii); and SS consumption and its possible repercussions on health and/or sports performance (iii). Regarding the use of SSs, the questionnaire recorded the general and current (during the sports season) consumption of SSs. It also included questions about what SSs they consumed, the time they consumed them (before, during, or after training and/or competition), and the consumption pattern (during training, during competition, both, rest season, or all year). Furthermore, the questionnaire included, among other questions: if the athletes followed a diet, who advised its consumption, what SSs did they consume, reasons for taking them, *n*, habitual place of purchase of the SSs, and the time of day when SSs were consumed.

### 2.3. Statistical Analysis

Quantitative variables are expressed as the mean value (M) ± standard deviation (SD), while qualitative variables are presented as frequencies and percentages. After checking for normality in the quantitative variables under the Shapiro−Wilk test, a Student’s t-test was performed to examine differences between the heavyweight and lightweight categories. For the variables that did not meet the normal distribution (lean mass (kg), number of weekly training sessions, consumption of medical SS supplement, consumption of SS in groups A and C), a Mann−Whitney U test was applied. Effect sizes (d) were calculated through Cohen’s d as: large (d > 0.8), moderate (d = 0.8 to 0.5), small (d = 0.5 to 0.2), and trivial (d < 0.2). Regarding the anthropometric assessments, these were only analyzed in the male rowers, because the limited sample of females (two female rowers in each bodyweight category) was too small for performing an interferential statistic. A χ2 test was used to compare the differences in consumption frequencies of each SS among heavyweight and lightweight athletes. The level of statistical significance was set as *p* < 0.05. Statistical analysis was performed using the SPSS statistical package (version 18.0).

## 3. Results

### 3.1. Descriptive Data, Analysis of Sports Practice, and Body Composition

Heavyweight rowers were older than lightweight rowers (24.2 ± 3.0 vs. 21.3 ± 2.3 years, *p* = 0.036, ES = 1.10), but no differences were observed in the number of weekly training sessions (13 ± 1 vs. 12 ± 3 sessions/week, *p* = 0.71). Regarding the anthropometric variables, male heavyweight rowers were heavier (*p* < 0.001, ES = 3.41), taller (*p* = 0.001, ES = 3.32), and reported higher fat (*p* = 0.009, ES = 1.72) and lean mass (*p* < 0.001, ES = 3.27) values. Nevertheless, there were no detected differences in body composition because any statistical differences were observed in % body fat (*p* = 0.104) or % lean body mass (*p* = 0.161) in male rowers (see Table 1).

### 3.2. Sport Supplement Consumption Data

According to our data, 40% of the rowers reported that they followed some type of diet. SSs were recommended by a medical doctor (25%), dietitian/nutritionist (12.5%), coach (12.5%), or self-regulated (50%). All rowers (100%) reported consuming at least one SS, with improvement of recovery between efforts (80%) and health reasons the most reported reasons among rowers. Medical doctors (50%) and rowers’ coaches (40%) were the main people who advised the consumption of SSs to the athletes. Although in some cases SSs were provided by the federation (20%) or by sponsors (5%), pharmacies (45%) were the most common place of purchase of SSs.

The different ranges of SSs ingested is reported in Table 2. Seventy-five percent of lightweight rowers consumed SSs during training and competition periods, whereas 25% only consumed SSs during the training period. Ninety-two percent of heavyweight rowers consumed SSs in both training and competition periods, and 7.7% of heavyweight rowers consumed SSs during the whole year, including rest season. The majority (92%) of heavyweight rowers consumed SSs before, during, and after exercise, however, 8% reported that they only consumed SSs after exercise. According to lightweights, 38% consumed SSs before, during, or after exercise, and 13% reported consuming SSs before or during exercise. The average consumption of SSs was higher in heavyweight rowers, but no statistical difference was observed with lightweight rowers (15.8 ± 5.6 vs. 11.6 ± 8.4, *p* = 0.188, ES = 0.66).

**Figure 1:**
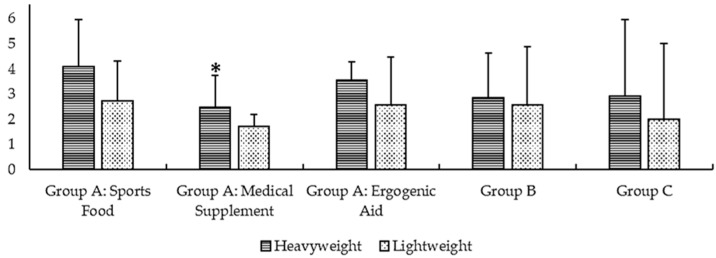
Number of supplements consumed in each category established by the Australian Institute of Sport (AIS) [20] by heavyweight and lightweight rowers. * Statistically significant differences between groups (*p* < 0.05).

**Figure 2:**
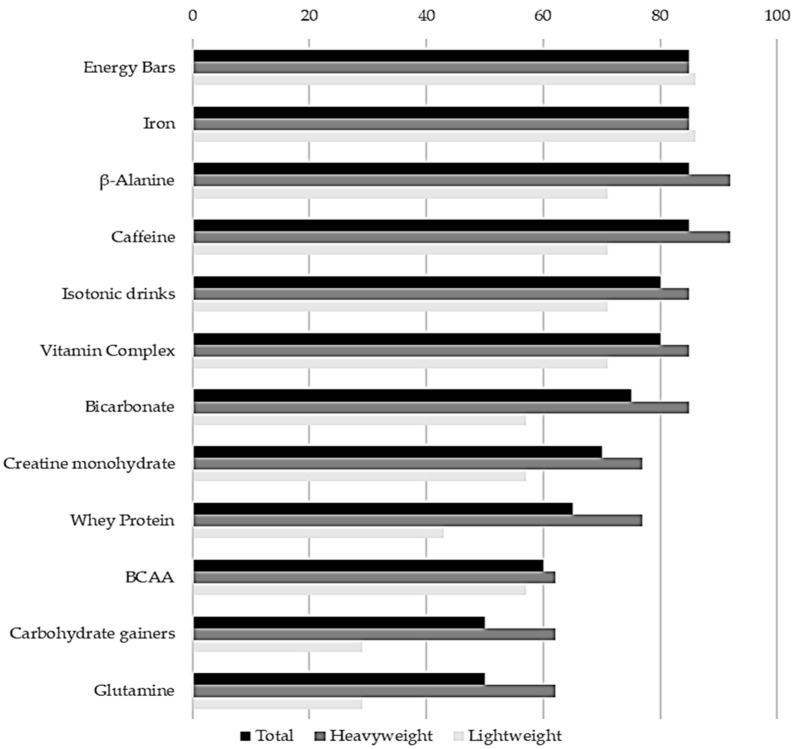
Type of sports supplements most used in total, heavyweight, lightweight rowers. Frequency refers to the percentage of players that used each type of sport supplement with respect to the number of participants who reported use of sports supplements. The sum of all percentages is >100% because there were players that used more than one sport supplement. BCAA = Branched Chain Amino Acids.

The average consumption of supplements per athlete was 62% higher in heavyweight rowers than in lightweight rowers (15.8 ± 5.6 vs. 11.6 ± 8.4 supplements). However, this difference was not statistically significant (*p* = 0.188). Heavyweight rowers reported a higher consumption of SSs in all of the categories identified by the Australian Institute of Sport (AIS) [20]. Furthermore, there was a trend of consuming more total supplements in group A (strong scientific evidence for sports scenarios) (10.1 ± 3.2 vs. 7.0 ± 3.8, *p* = 0.069, ES = 0.96), although statistically significant differences were observed only in the medical supplements (subgroup A) (2.5 ± 1.1 vs. 1.7 ± 0.5, *p* = 0.040, ES = 0.89). At the same time, there were no differences in group B (emerging scientific support, deserving of further research) (*p* = 0.776) and group C (scientific evidence not supportive of benefit and/or security amongst athletes) (*p* = 0.484) (see Figure 1). Figure 2 shows the type of sports supplements most used in total, heavyweight, lightweight rowers.

Energy and isotonic drinks were the most frequently consumed SSs in the sports food category (subgroup A) (85% and 80%, respectively). No significant differences were observed between weight groups, although heavyweight rowers had a higher consumption of gainers (62% vs. 29%), whey protein (77% vs. 43%), casein (23% vs. 0%), and beef protein (15% vs. 0%). In the medical supplements category (subgroup A), there was a trend towards greater vitamin D supplementation in heavyweight rowers (*p* = 0.07). A higher utilization rate of vitamin complexes and iron was also reported in the heavyweights, though the difference between groups was again not statistically significant (*p* > 0.05) (see Table 3).

In regard to the ergogenic aids category (subgroup A), a high consumption frequency of caffeine (85%), β-alanine (85%), sodium bicarbonate (75%), and creatine monohydrate (70%) was observed, without differences between heavyweight and lightweight rowers. Regarding the SSs of group B, it was observed that the most consumed were branched chain amino acids (BCAAs) (60%), glutamine (50%), carnitine (40%), and leucine (30%). There were no reported differences between rowers of different categories (*p* > 0.05). In the SSs of group C, a low utilization rate was observed, with relatively high consumption rates of melatonin (40%), arginine (35%), and green tea (30%) (see Table 3).

## 4. Discussion

To our knowledge, this is the first investigation that has analyzed the differences in anthropometric variables and SS consumption in elite rowers. A previous study in Olympic rowing athletes [16] reported a higher age (24.2 vs. 21.3 years, *p* = 0.036) and height (+ 6.8%, 1.88 vs. 1.76 m, *p* = 0.001) in heavyweight vs. lightweights rowers, which coincides with previous research [7]. Regarding body mass, the obtained mean values are similar to those obtained in the Olympic rowers (86.6 kg vs. 85.45 kg), while the weight of the lightweight rowers in the present sample is higher (71.6 kg vs. 65.5 kg) [16]. The greater body mass found in the lightweight rowers of our sample may be due to the fact that the data collection was conducted outside of the competition, although in this sense, the recommendations of the FISA must be taken into account; it recommends that the weight of these rowers never exceeds 5 kg in the 5–6 months prior, 3 kg in the 2−3 months prior, and 1 kg in the 24 h prior to competition, respectively [11]. Regarding body composition, there are no statistically significant differences in the percentage of fat mass between heavyweight and lightweight male rowers (15.1 ± 2.7% and 12.3 ± 2.1%). These results differ from those found in Olympic rowers, where there were differences between the two categories of weight [1]. Furthermore, the percentages of fat mass obtained were lower than those observed in high-performance rowers (20–23%) [5], which places them in adequate values with respect to endurance athletes [32].

Performance in 2000 m rowing has a strong association with the percentage of body fat [33], because higher levels of body fat can decrease economy and energy efficiency, thus affecting performance in 2000 m rowing [8]. These results indicate that international-level athletes present an adequate body composition during out of competition periods. They also show that in terms of body composition, heavyweight athletes do not present any hypothetical disadvantage [8] when compared to those in the lightweight category in economy or energy efficiency.

The prevalence of SS consumption greatly varies between sports due to multiple variables, among which sport modality or competition level stand out (39–100%) [17,18]. Due to the demand of this sport, the characteristics of the sample—elite international rowers—and the importance of nutrition in sports performance at these levels [5], it is logical that 100% of the sample consumes SSs. This supports the hypothesis of higher SS consumption at a higher level of competition [18]. The number of SSs consumed in our study by rowing athletes was higher in comparison to previous studies with sailors [29] and squash players [27]. However, lower values were reported when comparing bodybuilders that consume 20 SSs daily [28]. A high percentage of the rowers (75% of lightweight, 92.3% of heavyweight) consumed SSs in training and during the competition period. This is related to the SS most consumed by the sample, which mainly was used for training adaptations, improvement of sport performance in competitions, recovery post-effort, or to prevention of nutritional deficits.

Concerning the consumption of SSs from the sports food subgroup, a high percentage of energy drinks (85%) and isotonic (80%) consumption was observed, similar to data previously reported in elite athletes [17], although consumption was higher than those found in international Spanish athletes from other sports modalities [27]. The consumption of these SSs could be suitable to maintain high training volumes and enhance the recovery process [34]. Consumption of carbohydrate supplements with added protein (classified as gainers) was higher in the present sample (50%) than in previous studies in elite Spanish athletes (15%) [35]. In contrast, the consumption prevalence of supplements like whey protein (65%), casein (15%), and meat protein (10%) agreed with similar studies in elite Spanish athletes of different sports modalities [27,35]. It could be reasoned that the post-exercise consumption of gainers could be better than the consumption of isotonic drinks because supplementation with carbohydrates in conjunction with long-term proteins has a positive effect on performance in exhaustion tests with respect to the exclusive intake of carbohydrates [36]. Furthermore, in cases of suboptimal consumption of carbohydrates in the post-exercise recovery phase, the addition of proteins increases glycogen re-synthesis and reduces muscle damage symptoms [37]. Finally, high levels of quality protein consumption (90% between whey, casein, and beef protein supplements) could explain the appropriated body composition values observed in our sample, increasing muscle protein synthesis and helping to maintain a nitrogen balance status [38].

With respect to the consumption of medical supplements, high consumption of iron supplements (85%) and vitamin complexes (80%) were reported. However, differences in consumption between multiple groups were not statistically significant. The data of our study reported higher values in comparison to those found with elite athletes from other modalities [27,35]. The high iron consumption may be due to prevention and treatment, since iron deficiency is the most prevalent nutritional deficiency in athlete populations, especially in women [39]. Moreover, this nutritional deficiency may present a prevalence of 27% in finishing the competitive period in rowing [40]. The consumption of vitamin supplements is similar to other investigations in elite rowers (84%) [41], but greater than other studies in elite athletes from different sports modalities (35–50%) [27,35]. This may be due to the possible use of these supplements as antioxidants, since there is a correlation between antioxidant status and performance in competitive rowers [42].

In reference to group A, ergogenic aids, a high consumption of β-alanine was observed (85%). β-alanine supplementation increases muscle carnosine levels due to pH regulation and the improvement of muscle contractility [43]. β-alanine tends to improve performance in a 2000 m test of high-performance rowers [44,45]. Therefore, its use reflects a potential performance-enhancing effect in the sample. In addition, a possible greater effect on performance has been observed when combining sodium bicarbonate supplementation in a 2000 m test of rowers [44]. The consumption of sodium bicarbonate was 75% of the sample, whereas 100% consumed β-alanine. These results suggest a good selection of alkalizing supplements, because the co-ingestion of sodium bicarbonate and β-alanine resulted in probable (2.6% (90% CL ± 1.5%)) and very probable performance improvements (1.4% (90% CL ± 1.2%)), respectively [46]. Caffeine supplementation—one of the most popular pre-conditioning strategies in rowing—had a high rate of consumption (85%), which agreed with previous studies [46]. On the one hand, previous investigations have not reported an ergogenic effect of caffeine supplementation in a 2000 m test [47], on the other hand, other studies observed higher mean power in a six minute test [48], 2000 m test [49], or in a test of four sets of 2000 m [50] after caffeine supplementation in trained rowers. The observed consumption of creatine supplementation was 70%. Creatine supplementation has demonstrated improved performance in a 2000 m test [51], and it could favor adaptations to training due to its ability to accelerate recovery processes and the intensity of training sessions [52].

The most consumed SSs in group B were BCAAs (60%), glutamine (50%), and carnitine (40%), with no differences found between rowers of different weight categories (*p* > 0.05). These results are higher when compared to other studies with low levels of scientific evidence that had consumptions of 8–38% BCAAs and 18% other SSs [27,35,53]. However, one study has observed diminishing phosphorus levels in the blood due to BCAAs and glutamine supplementation during the post-exercise recovery of a 2000 m rowing test. Nevertheless, the groups supplemented with glutamine alone seemed to have a lower concentration of CK in the blood than the rest, suggesting positive effects in reducing fatigue factor stimulation [54]. Additionally, other references indicated that glutamine supplementation could be useful in improving immune function and the post-exercise inflammatory defense reaction [54,55,56].

A moderate vitamin C intake (45%) was reported in the sample, contrasting with other studies that reported a consumption of ≤5% in elite athletes [27,35]. However, the sample was similar to a previous study carried out with professional endurance athletes [53] and others performed on elite rowers [42] that saw a moderate positive correlation between the distance covered in the performance test and the consumption of vitamin C [42].

The total consumption of SSs in group C was considerably lower than those in group A, and although like the total in group B, their consumption was more diversified (eight SSs in group B vs. 18 SSs in group C). Among the different SSs in group C, the most consumed SSs were melatonin (40%), arginine (35%), and green tea (25%). The rest had a consumption of ≤25%. SS consumption continues to increase among elite athletes [41] due to a perceived need for supplementation [57], especially in elite rowers, as was found in this study. However, it is necessary to educate athletes on the use of SSs by promoting educational programs on having the appropriate combinations and doses of supplements in order to minimize the possible health risks caused by inappropriate use [57].

The current investigation has several limitations that should be discussed to enhance its applicability to real sports contexts. First, the reduced number of participants made it difficult to detect statistical differences, and reduces the statistical power of the analysis performed. Regarding the sample size, another limitation is the reduced number of female participants in each category (two participants in heavyweight/lightweight, respectively), making it impossible to carry out an inferential analysis on female participants. Nevertheless, this limitation at the statistical level is based on the characteristic of the sample, because it was all of the members of the Spanish National Rowing Team, one of the national teams with a higher number of participants in international rowing competitions (Olympic games, World Championship, and European Championship), so these results could be very useful for other international rower teams. Second, although the method used to determine body composition is not the gold standard [58], the electrical bioimpedance used in this study has reported good accuracy for detecting body composition compared to anthropometric [21] and DXA methods [22].

## 5. Conclusions

According to anthropometric factors, heavyweight rowers have relatively similar body compositions to lightweight rowers. In relation to SSs, the consumption of at least one SS in all categories analyzed (heavyweight/lightweight) has been observed. No differences were reported in SS consumption between rowing categories except for medical supplements (higher in the heavyweight group). Nevertheless, it is possible to improve the ergogenic plans of this elite sample; thus, sport supplement consumption needs to be individualized, through the recommendation of a diet/nutrition specialist, and based on the criteria of safety, efficacy, and legality.

## Figures and Tables

**Table 1:** Age and anthropometric variables in heavyweight and lightweight rowers divided by sex. Data are presented as M ± SD.

Variable	Total Rowers		Female Rowers		Male Rowers		*p*-Value (Male Rowers)	ES (Male Rowers)
	Hw	Lw	Hw	Lw	Hw	Lw		
Weight (kg) *	86.6 ± 7.1	71.6 ± 6.3	74.5 ± 2.1	62.8 ± 2.3	88.8 ± 4.9	75.2 ± 1.9	<0.001	3.41
Height (m) *	1.88 ± 0.06	1.76 ± 0.05	1.78 ± 0.06	1.69 ± 0.00	1.90 ± 0.04	1.79 ± 0.02	<0.001	3.32
BMI (kg/m^2^) *	24.5 ± 1.2	23.0 ± 0.9	23.5 ± 0.1	22.0 ± 0.8	24.7 ± 1.2	23.4 ± 0.7	0.042	1.29
Body fat (kg) *	14.3 ± 3.5	10.2 ± 2.2	19.1 ± 1.1	12.6 ± 2.0	13.5 ± 3.0	9.2 ± 1.6	0.009	1.72
Lean Body Mass (kg) *	72.3 ± 8.2	61.4 ± 8.0	55.4 ± 4.0	50.2 ± 0.4	75.4 ± 3.3	66.0 ± 2.4	<0.001	3.27
Fat Mass (%)	16.8 ± 4.67	14.5 ± 4.2	25.7 ± 0.2	20.0 ± 2.4	15.1 ± 2.7	12.3 ± 2.1	0.104	1.18
Lean Body Mass (%)	83.3 ± 4.7	85.5 ± 4.2	74.4 ± 0.2	80.0 ± 2.4	84.9 ± 2.7	87.7 ± 2.1	0.161	1.18

Statistical analysis was only performed on male rowers; Hw = heavyweight; Lw = lightweight; BMI = body mass index. * Statistically significant differences between groups (*p* < 0.05).

**Table 2:** Overall number and percentage of SSs ingested by heavyweight and lightweight rowers.

Number of SS Ingested	Overall		Heavyweight		Lightweight	
	*n*	%	*n*	%	*n*	%
<5 SS/day	2	10	0	0	2	29
>5 <10 SS/day	2	10	1	8	1	14
>10 <15 SS/day	5	25	4	31	1	14
≥ 15 SS/day	11	55	8	62	3	43

SS = sport supplement.

**Table 3:** SS consumption of heavyweight and lightweight rowers in the specific categories by the AIS [20].

Category		Supplements	Overall		Heavyweight		Lightweight		*p*-Value
			*n*	%	*n*	%	*n*	%	
**Group A**	Sport Foods	Energy Bars	17	85	11	85	6	86	1.000
		Isotonic drinks	16	80	11	85	5	71	0.587
		Whey Protein	13	65	10	77	3	43	0.174
		Carbohydrate gainers	10	50	8	62	2	29	0.350
		Electrolytes	9	45	6	46	3	43	1.000
		Casein	3	15	3	23	0	0	0.521
		Maltodextrin	2	10	2	15	0	0	0.521
		Beef Protein	2	10	2	15	0	0	0.521
	Medical Supplements	Iron	17	85	11	85	6	85.7	1.000
		Vitamin Complex	16	80	11	85	5	71	0.587
		Vitamin D	9	45	8	62	1	14	0.070
		Mineral Complex	2	10	2	15	0	0	0.521
	Ergogenic Aids	β-Alanine	17	85	12	92	5	71	0.270
		Caffeine	17	85	12	92	5	71	0.270
		Bicarbonate	15	75	11	85	4	57	0.290
		Creatine monohydrate	14	70	10	77	4	57	0.613
		Creatine alkaline	1	5	1	8	0	0	1.000
**Group B**		BCAA	12	60	8	62	4	57	1.000
		Glutamine	10	50	8	62	2	29	0.350
		Vitamin C	9	45	6	46	3	43	1.000
		L carnitine	8	40	4	31	4	57	0.356
		Leucine	6	30	5	39	1	14	0.354
		Fatty Acids ω-3	5	25	3	23	2	29	1.000
		Vitamin E	4	20	3	23	1	14	1.000
		Acetil L carnitine	1	5	0	0	1	14	0.350
**Group C**		Melatonin	8	40	7	54	1	14	0.158
		Arginine	7	35	5	39	2	29	1.000
		Green tea	6	30	4	31	2	29	1.000
		Pre-Workout	5	25	5	39	0	0	0.114
		Taurine	4	20	2	15	2	29	0.587
		Zinc	3	15	2	15	1	14	1.000
		Fatty acids ω-6	2	10	2	15	0	0	0.521
		Fatty acids ω-9	2	10	2	15	0	0	0.521
		Essentials amino acids	2	10	1	8	1	14	1.000
		Citrulline malate	2	10	1	8	1	14	1.000
		Spirulina	2	10	2	15	0	0	0.521
		Ginseng	2	10	2	15	0	0	0.521
		Guaraná	2	10	1	8	1	14	1.000
		Magnesium	2	10	1	8	1	14	1.000
		Chitosan	1	5	0	0	1	14	0.350
		Collagen	1	5	0	0	1	4	0.474
		Royal Jelly	1	5	1	8	0	0	1.000
		Vitamin K	1	5	0	0	1	14	0.350
